# Improved predictive ability of the Montreal Cognitive Assessment for diagnosing dementia in a community-based study

**DOI:** 10.1186/s13195-015-0156-8

**Published:** 2015-11-09

**Authors:** Jung-Lung Hsu, Yen-Chun Fan, Ya-Li Huang, Jui Wang, Wei-Hung Chen, Hou-Chang Chiu, Chyi-Huey Bai

**Affiliations:** Graduate Institute of Humanities in Medicine, Taipei Medical University, Taipei, Taiwan; Brain and Consciousness Research Center, Taipei Medical University, Shuang Ho Hospital, New Taipei City, Taiwan; Section of Dementia and Cognitive Impairment, Department of Neurology, Chang Gung Memorial Hospital, Linkou, Taiwan; School of Public Health, College of Public Health and Nutrition, Taipei Medical University, 250 Wu-Hsing Street, Taipei City, 110 Taiwan; Department of Public Health, College of Medicine, Taipei Medical University, 250 Wu-Hsing Street, Taipei City, 110 Taiwan; Institute of Epidemiology and Preventive Medicine, College of Public Health, National Taiwan University, Taipei, Taiwan; Department of Neurology, Shin Kong Wu Ho-Su Memorial Hospital, Taipei, Taiwan

## Abstract

**Introduction:**

We compared the predictive ability of the Mini-Mental State Examination (MMSE) and the Montreal Cognitive Assessment (MoCA) to diagnose dementia in a community-based study.

**Methods:**

A total of 276 people aged 60 years or older were enrolled. All of the participants were administered face-to-face interview questionnaires and MoCA and MMSE examinations. The receiver operating characteristic curve method and area under curve were performed to assess the predictive ability for diagnosing dementia.

**Results:**

The 276 participants had a mean age of 67.9 ± 6.1 years and mean education duration of 11.4 ± 4.0 years. In general, the MoCA yielded higher AUCs (0.891) with favorable sensitivity (78 %) and excellent specificity (94 %) compared with the MMSE in differentiating the participants with and without dementia in either the total sample or all subgroups.

**Conclusion:**

Our study determined a higher predictive ability in the MoCA than in the MMSE for diagnosing dementia according to *Diagnostic and Statistical Manual of Mental Disorders, Fourth Edition* (DSM-IV) criteria in a community-based sample with a broader range of education level.

## Introduction

The population is rapidly aging worldwide [1, 2]. The prevalence of dementia increases rapidly with age and leads to a burden on public health [3–7]. Detecting moderate cognitive dysfunction early could decrease the risk factors associated with vascular events, resulting in the prevention of dementia [8].

Cognitive function can be measured using several instruments, such as the Clinical Dementia Rating Scale, the Global Deterioration Scale, and the Mini-Mental State Examination (MMSE) [9–12]. The MMSE has been broadly applied to assess cognitive function [13–16], but it is insufficient and highly influenced by education [17]. The Montreal Cognitive Assessment (MoCA) is a new instrument for detecting mild cognitive dysfunction with superior sensitivity and can be completed in 10 minutes by clinicians [18].

Numerous studies have validated the cognitive screening ability between the MoCA and MMSE, showing that the MoCA is a more useful screening instrument than the MMSE for detecting dementia [19–24]. Some researchers, however, have indicated that the MoCA was not superior to the MMSE for assessing patients with mild cognitive impairment (MCI) [25, 26]. In some hospital-based research, the gold standards were the neuropsychological tests [19–23]. Some studies demonstrated their findings in the community, but did not choose a gold standard of the *Diagnostic and Statistical Manual of Mental Disorders, Fourth Edition* (DSM-IV) [24]. To decide which is best (i.e., diagnosed by physicians according to DSM-IV criteria and community-based samples) is difficult. Thus, the conclusions reported to date remain obscure.

The purpose of this study was to compare the screening ability of the MoCA with that of the MMSE for detecting dementia in a community-based population. In addition, we performed an analysis of subgroups stratified according to age, sex, and education level to examine the predictive ability of the MoCA for diagnosing dementia.

## Methods

### Study population

The participants were randomly sampled in 2007–2008 from among all of the residents aged 60 years or older in a community (*n* = 448) that neighbored a teaching hospital. They were invited to participate in a community-based prospective cohort study investigating the cardiovascular and cerebrovascular risk factors in the general population [27, 28]. The participants were invited again after 3 years to undergo follow-up examinations to measure the neurological assessments from 2011 to 2013. Those who declined to participate in the follow-up study were excluded. A total of 276 remaining individuals were enrolled in this study for the analysis.

### Instruments

The MoCA scale is a cognitive screening test with a high level of interrater reliability and internal consistency for detecting dementia. It can be administered in approximately 10 minutes [18, 20, 22]. Eight subitems were used to measure cognitive function: (1) visuospatial/executive function (an alternation task adapted from the Trail Making Test Part B task, a clock-drawing task, and a three-dimensional cube copy), (2) naming (a three-item confrontation naming task with low-familiarity animals), (3) memory (the short-term memory recall task with two learning trials of five nouns, no points), (4) attention (a sustained attention task with target detection using tapping, a serial subtraction task, and digits forward and backward), (5) language (repetition of two syntactically complex sentences and a phonemic fluency task), (6) abstraction (a two-item verbal abstraction task), (7) delayed recall (5-minute delayed memory assessment of the short-term memory recall task for five nouns), and (8) orientation (evaluated time and place) [18]. For the participants with less than 12 years of education, the final score was the MoCA plus 1 point. The interrater intraclass correlation coefficient of MoCA in this study was 0.852, which showed high reliability.

The MMSE test has adequate interrater reliability and internal consistency in predicting dementia. It is used to screen for cognitive impairment and dementia [12].

### Clinical evaluations

The participants were recruited by performing two-stage screening. During the first stage in 2007–2008, a survey with a standardized and structured questionnaire was conducted by well-trained interviewers to obtain demographic information. Age (as a continuous variable and divided into three categories: 60–69, 70–79, and ≥80 years), sex, and duration of education (as continuous variable and divided into three categories: ≤6, 7–12, and >12 years) were evaluated. An approximately 2-year follow-up survey was conducted to collect the results from a clinical examination performed by neurologists. From 2011 to 2013, all of the participants were administered face-to-face interview questionnaires and the MoCA and MMSE examinations in approximately 30 minutes in clinical practice. The clinical diagnosis of dementia was based on the judgment of a single neurologist. The neurologist made the diagnosis on the basis of interviews of participants and their caregivers (usually the participant’s spouse). The participants’ previous function was evaluated on the basis of caregivers’ and participants’ statements. Details were collected regarding cognitive function, such as memory, communication, motor function, recognition or identification of objects, and executive function. Daily activities, including shopping, housekeeping, cocking, doing laundry, and using transportation, were also examined. Next, the office-based neurological examination was performed. If the participant had memory impairment and other domains of cognitive impairment based on the DSM-IV diagnostic criteria for Alzheimer’s disease, dementia was diagnosed. The laboratory examination included a blood test, thyroid function examination, and liver and renal function. Brain computed tomography was performed to exclude structural lesions for the possibility of secondary dementia. The neurologist was blinded to participants’ cognitive function state.

### Statistical analysis

Statistical analysis was performed using SAS version 9.2 software (SAS Institute, Cary, NC, USA). The descriptive results of continuous variables were expressed as mean ± standard deviation. Baseline demographic status and MoCA scores were compared by using a *t* test or one-way analysis of variance for continuous variables and the χ^2^ test for categorical variables. A *P* value less than 0.05 was considered statistically significant.

A receiver operating characteristic (ROC) curve was constructed to establish MoCA cutoff points for all of the participants and subgroups. The area under the curve (AUC) was calculated for each of the ROC curves with a 95 % confidence interval. The cutoff scores were derived from the ROC coordinate points, at which both sensitivity and specificity were calculated using Youden’s J statistic (Youden’s index).

All participants provided written informed consent before study entry. This study was also evaluated and approved by the institutional review board of Taipei Medical University and Shin Kong Wu Ho-Su Memorial Hospital.

## Results

Among the 276 participants, 16 (5.8 %) were diagnosed with dementia and 260 (94.2 %) were diagnosed with normal cognitive function. The demographic variables and MoCA scores of all 276 participants and the subgroups were stratified according to sex (Table 1). Compared with the women, the men had a significantly higher duration of education and were older. The MoCA scores for visuospatial function, naming, and abstraction of the men were significantly higher than those of the women. However, no differences in total score by sex were observed.

**Table 1:** Comparison of demographic variables and the MoCA scale stratified by gender

	Total	Gender		
		Male	Female	
Variables	*N =* 276	*N =* 136	*N =* 140	P value ^a^
Age (years ± SD)	67.93 ± 6.06	68.93 ± 6.30	66.95 ± 5.68	0.0064^**^
Male (%)	136 (49.28%)			
Education (years ± SD)	11.38 ± 4.01	12.39 ± 3.98	10.40 ± 3.82	< 0.0001^***^
Total Score	25.33 ± 3.44	25.51 ± 3.24	25.16 ± 3.64	0.4088
Visuospatial	3.97 ± 1.03	4.15 ± 0.97	3.81 ± 1.06	0.0057^**^
Naming	2.76 ± 0.57	2.90 ± 0.35	2.63 ± 0.69	< 0.0001^***^
Memory1	4.03 ± 1.06	3.98 ± 1.07	4.08 ± 1.04	0.4418
Memory2	4.61 ± 0.81	4.55 ± 0.84	4.67 ± 0.77	0.3364
Attention1	1.85 ± 0.40	1.85 ± 0.36	1.84 ± 0.44	0.8334
Attention2	0.95 ± 0.22	0.94 ± 0.24	0.96 ± 0.20	0.5473
Attention3	2.85 ± 0.42	2.85 ± 0.41	2.84 ± 0.44	0.8441
Language1	1.25 ± 0.73	1.24 ± 0.73	1.26 ± 0.73	0.7415
Language2	0.80 ± 0.40	0.76 ± 0.43	0.84 ± 0.37	0.1025
Abstraction	1.30 ± 0.76	1.43 ± 0.75	1.19 ± 0.76	0.0086^**^
Delayed Recall	3.04 ± 1.71	2.98 ± 1.73	3.11 ± 1.70	0.5321
Orientation	5.95 ± 0.24	5.94 ± 0.24	5.95 ± 0.25	0.7632

*MoCA* Montreal Cognitive Assessment, *SD* standard deviation

*: *P* < 0.05; **: *P* < 0.01; ***: *P* < 0.0001

^a^. Tested by two-sample *t* test

As shown in Table 2, the MoCA scale scores were stratified according to education level and age. Regarding education level, significant differences were observed between three groups in total scores and the scores for eight subitems.

**Table 2:** Comparison of demographic variables and the MoCA scale stratified by education level and age

	Education					Age (years)				
	Low	Middle	High			60–69	70–79	≧ 80		
Variables	*N =* 61	*N =* 118	*N =* 97	P value ^a^	*P*_trend_	*N =* 171	*N =* 91	*N =* 14	*P* value ^a^	*P*_trend_
Age (years ± SD)	67.77 ± 5.63	68.58 ± 6.59	67.23 ± 5.62	0.2572	0.4378	63.96 ± 2.91^a^	73.27 ± 2.79^b^	81.57 ± 2.44^c^	< 0.0001^***^	< 0.0001^***^
Male (%)	20 (32.79%)	53 (44.92%)	63 (64.95%)	0.0002^**^		77 (45.03%)	48 (52.75%)	11 (78.57%)	0.0392^*^	
Education (years ± SD)	5.66 ± 1.35^a^	10.74 ± 1.53^b^	15.76 ± 1.21^c^	< 0.0001^***^	< 0.0001^***^	11.52 ± 4.10	11.16 ± 3.96	11.00 ± 3.33	0.7403	0.4450
Total Score	23.39 ± 3.87^a^	25.28 ± 3.38^b^	26.62 ± 2.58^c^	< 0.0001^***^	< 0.0001^***^	25.94 ± 3.09^a^	24.62 ± 3.66^b^	22.64 ± 4.09^b^	0.0001^**^	< 0.0001^***^
Visuospatial	3.38 ± 1.08^a^	3.93 ± 1.02^b^	4.40 ± 0.77^c^	< 0.0001^***^	< 0.0001^***^	4.03 ± 1.00	3.95 ± 1.02	3.50 ± 1.34	0.1696	0.1116
Naming	2.59 ± 0.78^a^	2.74 ± 0.55	2.90 ± 0.37^b^	0.0032**	0.0007^**^	2.80 ± 0.52	2.70 ± 0.62	2.64 ± 0.74	0.3096	0.1281
Memory1	3.62 ± 1.22^a^	4.07 ± 1.04^b^	4.25 ± 0.89^b^	0.0022**	0.0008^**^	4.15 ± 1.02	3.86 ± 1.06	3.50 ± 1.31	0.0269^*^	0.0072^**^
Memory2	4.41 ± 0.95^a^	4.57 ± 0.76	4.80 ± 0.73^b^	0.0405*	0.0117^*^	4.64 ± 0.80^a^	4.64 ± 0.67^a^	4.10 ± 1.37^b^	0.1198	0.1813
Attention1	1.80 ± 0.44	1.82 ± 0.45	1.91 ± 0.29	0.1816	0.0855	1.88 ± 0.36^a^	1.86 ± 0.38^a^	1.43 ± 0.65^b^	0.0002^**^	0.0050^*^
Attention2	0.92 ± 0.28	0.95 ± 0.22	0.97 ± 0.17	0.3658	0.1602	0.96 ± 0.20^a^	0.95 ± 0.23	0.86 ± 0.36	0.2435	0.1686
Attention3	2.80 ± 0.48	2.84 ± 0.45	2.89 ± 0.35	0.4668	0.2186	2.87 ± 0.38	2.79 ± 0.51	2.93 ± 0.27	0.2670	0.4875
Language1	0.92 ± 0.78^a^	1.18 ± 0.74^a^	1.55 ± 0.56^b^	< 0.0001^***^	< 0.0001^***^	1.35 ± 0.68^a^	1.15 ± 0.80	0.71 ± 0.47^b^	0.0022^**^	0.0008^**^
Language2	0.84 ± 0.37	0.75 ± 0.43	0.85 ± 0.36	0.1927	0.6787	0.86 ± 0.35^a^	0.75 ± 0.44	0.50 ± 0.52^b^	0.0011^**^	0.0004^**^
Abstraction	0.79 ± 0.76^a^	1.26 ± 0.76^b^	1.68 ± 0.55^c^	< 0.0001^***^	< 0.0001^***^	1.40 ± 0.72^a^	1.11 ± 0.82^b^	1.36 ± 0.74	0.0115^*^	0.0297^*^
Delayed Recall	2.49 ± 1.76^a^	2.94 ± 1.67^a^	3.52 ± 1.63^b^	0.0008^**^	0.0002^**^	3.27 ± 1.63^a^	2.73 ± 1.81^b^	2.35 ± 1.74	0.0149^*^	0.0039^**^
Orientation	5.89 ± 0.37	5.96 ± 0.20	5.97 ± 0.17	0.0828	0.0472^*^	5.95 ± 0.21^a^	5.97 ± 0.18^a^	5.71 ± 0.61^b^	0.0010^*^	0.0560

*MoCA* Montreal Cognitive Assessment, *SD* standard deviation

*: *P* < 0.05 ; **: *P* < 0.01 ; ***: *P* < 0.0001

^a^. Tested by ANOVA with Scheffe’s test and χ^2^ test

**Fig. 1:**
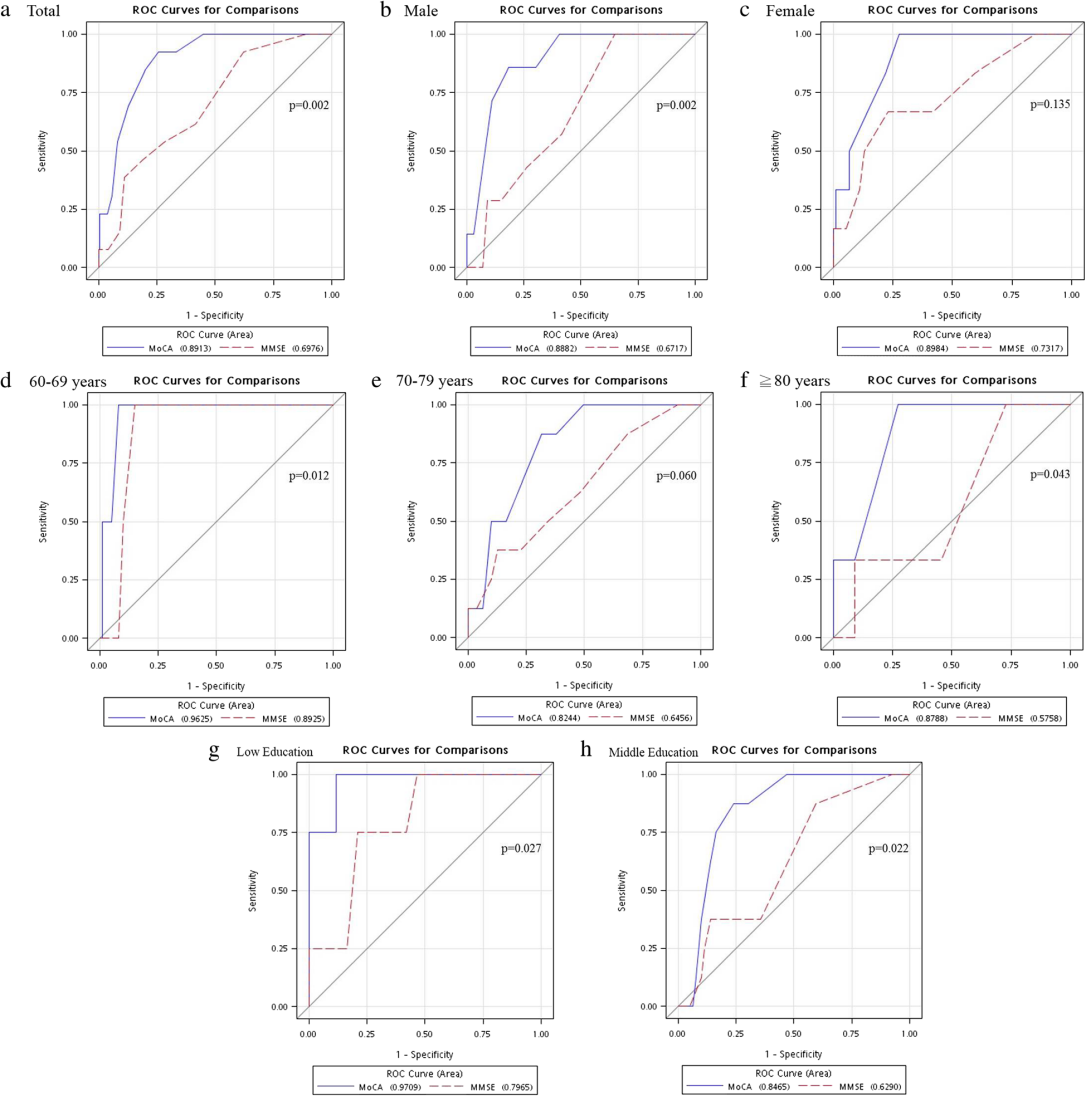
Receiver operating characteristic (ROC) curves constructed after assessing dementia with the Montreal Cognitive Assessment (MoCA) and the Mini-Mental State Examination (MMSE). The ROC curves were tested in the total sample and in stratified subgroups according to the age, sex, and education level. **a** Total, **b** men, **c** women, **d** 60 men, ted and **e** ages 70–79 years, **f** age ≥80 years, **g** low education, and **h** middle education groups.

ROC analysis results for dementia are shown in Table 3 and Fig. 1. For all of the 276 participants, the MoCA yielded greater AUCs than the MMSE to differentiate between participants with and without dementia (0.8913 vs 0.6976). In addition, the MoCA in all subgroups achieved higher AUCs than did the MMSE for dementia. Because no dementia patients were in the high-education group, AUCs could not be calculated.

**Table 3:** AUC and cut-off points of the MoCA, MMSE stratified by gender, education level, and age

	MoCA					MMSE				
	Area	95% CI	Optimal cut-off	Sensitivity	Specificity	Area	95% CI	Optimal cut-off	Sensitivity	Specificity
Total	0.8913	0.83–0.96	23.5	0.78	0.94	0.6976	0.56–0.83	28.5	0.38	0.92
Gender										
Men	0.8882	0.79–0.98	22.5	0.84	0.88	0.6717	0.51–0.84	28.5	0.35	1.00
Women	0.8984	0.81–0.99	23.5	0.76	1.00	0.7317	0.50–0.96	25.5	0.77	0.67
Age (years)										
60–69	0.9625	0.90–1.00	23.5	0.84	1.00	0.8925	0.83–0.96	25.5	0.85	1.00
70–79	0.8244	0.71–0.94	23.5	0.67	0.88	0.6456	0.44–0.85	24.5	0.87	0.38
≧ 80	0.8788	0.69–1.00	22.0	0.73	1.00	0.5758	0.19–0.96	28.5	0.27	1.00
Education level										
Low	0.9709	0.91–1.00	20.5	0.89	1.00	0.7965	0.60–0.99	24.5	0.79	0.75
Middle	0.8465	0.75–0.94	23.5	0.80	0.90	0.6290	0.45–0.81	28.5	0.41	0.88
High			22.5	0.92	1.00			27.5	0.63	1.00

*CI* Confidence interval

We determined the cutoff points of the MoCA scale for all participants. The cutoff score on the MoCA was 23.5, demonstrating favorable sensitivity (78 %) and specificity (94 %).

## Discussion

We show that the MoCA had superior predictive ability for detecting dementia. The sensitivity of 0.78 and specificity of 0.94 of MoCA were higher than the values for the MMSE. For the subgroup analysis, the findings were essentially the same for the women, younger participants (i.e., aged 60–69 years), and those with a low education level. The optimal cutoff score on the MoCA was 23.5, with a high AUC of 0.89.

In most previous studies [19–24], researchers have also reported that the MoCA appeared to be more useful as a cognitive screening tool than the MMSE. However, the investigators in these studies have not used the same criterion of cognitive dysfunction. The study samples may have been recruited primarily from a clinic and not from the general population, as in our present study [19–23]. In only one study did researchers recruit community-based participants, but the diagnostic criteria were not based on DSM-IV [24]. Other researchers have reported that the MoCA was not more effective than the MMSE in assessing dementia [25, 26]. The inconsistent findings might be due to the sample location, different participant ages, and varying criteria for diagnosing cognitive function. Distinct sample distributions in different studies make comparing findings difficult.

The cutoff score on the MoCA analyzed for our participants was 23.5, which is consistent with previous studies that indicated a high sensitivity and specificity for identifying Alzheimer’s disease and dementia, with optimal cutoff scores ranging from 22 to 26 [19–24]. In some studies, researchers have presented a cutoff point of 23 for the MoCA, similar to our findings, with the average education duration ranging from 9.8 to 14.4 years [19, 22, 24]. This means that the MoCA score was not significantly influenced by education level within this range. Furthermore, our results are similar to those reported by Tsai et al., who studied a Taiwanese population [22]. They reported that the optimal cutoff point for dementia was 23 or 24, with a sensitivity of 92 % and specificity of 78 %. These similar results may reflect that both sample populations were close to coherent and the criteria for identifying cognitive impairment were remarkably similar.

In the subgroup analysis, age, sex, and education level were stratified according to the aforementioned definition. The results were largely unchanged. Consistent with previous studies [29, 30], the female participants exhibited slightly higher AUCs. As expected, this suggests that women received shorter education than men did, according to their cultural background in China [31]. High MoCA scores were observed for the younger participants and those with a high education level. This result was similar to outcomes in a Chinese-American study [31]. Education and age could influence the MoCA score.

The results for the eight subitems of the MoCA differed in the subgroups of age, sex, and education level. Nonetheless, few studies have investigated the association between subgroups and eight subitems [32, 33]. High scores for visuospatial function, naming, and abstraction among the men were determined in our study. However, this result was not consistent with findings in an Italian study, in which researchers showed that sex influenced the attention and memory domains [32]. The culture difference may explain the disparity in the contrasting results. Our findings regarding the subitem scores of the older participants are comparable to the Italian data, except for the domains of attention and orientation [32]. Another Taiwanese study, published in 2012, showed that the subitem scores on the MoCA were influenced by education level. Participants with 0–6 years of education had the lowest scores for visuospatial and executive function, abstraction, orientation, and naming domains [33].

The strengths of the present study are the use of a community-based population, DSM-IV criteria for diagnosing dementia based on clinical assessments, and analysis of subgroups stratified according to age, sex, and education level. Previous hospital-based sample studies [19–23] led to selection bias, and thus their results cannot be extrapolated to the general population. Dementia was diagnosed by a neurologist according to DSM-IV criteria, and the accuracy was high. Other studies have applied questionnaires to define dementia. Subgroup analysis was also performed in the present study. The results were similar and showed that the MoCA was a stable instrument for assessing dementia. The prevalence of dementia in our study was comparable (5.8 %) to that in other studies (5.7 % and 5.4 %) done in Taiwan [34, 35]. The representativeness of this community-based sample is supported.

Several limitations should be carefully addressed. First, the participants were recruited from one district in Taiwan. The representativeness was limited and might not be generalizable to other regions. However, the participants were randomly selected in proportions that were consistent within the population. The discrepancy was then minimized. Second, a substantial proportion of the initially enrolled participants withdrew from the study during the follow-up period. Nevertheless, the difference in demographic distribution between the analyzed sample and those who withdrew was not significant. Third, there were no dementia events in the high education level group, which might have influenced the MoCA cutoff score. Nonetheless, it was similar to that in another Taiwanese study with a MoCA cutoff score of 23/24. Finally, MCI was not assessed in the present study. Diagnoses of MCI are very heterogeneous due to different diagnostic criteria. On the basis of the National Institute on Aging/Alzheimer’s Association core clinical criteria for MCI [36], the diagnosis of MCI requires detailed episodic memory measures, such as the Free and Cued Selective Reminding Test or the Logical Memory subtest of the Wechsler Memory Scale. Our study did not include this measurement. A future study will be focused on the relationship between the MoCA score and participants with MCI.

## Conclusions

This study demonstrates that the MoCA had a superior capability to detect dementia compared with the MMSE in a community-based sample with a broad range of education levels. Dementia was diagnosed by a physician according to DSM-IV criteria. Our results not only suggest that the MoCA is a useful screening tool but also explain the different features in the stratified groups. Future studies should be focused on early detection and treatment of cognitive dysfunction in clinical practice.

## Abbreviations

AUC: area under the curve
DSM-IV: *Diagnostic and Statistical Manual of Mental Disorders, Fourth Edition*
MCI: mild cognitive impairment
MMSE: Mini-Mental State Examination
MoCA: Montreal Cognitive Assessment
ROC: receiver operating characteristic
